# Improving the pre-screening of eligible patients in order to increase enrollment in cancer clinical trials

**DOI:** 10.1186/s13063-014-0535-7

**Published:** 2015-01-16

**Authors:** Boris Campillo-Gimenez, Camille Buscail, Oussama Zekri, Brigitte Laguerre, Elisabeth Le Prisé, Renaud De Crevoisier, Marc Cuggia

**Affiliations:** Inserm U1099 - LTSI, Equipe Données Massives en Santé - Université Rennes 1, 2 rue du Professeur Léon Bernard, 35043 Rennes Cedex, France; Department of Medical Information, University Hospital of Rennes, 2 rue Henri Le Guilloux, 35000 Rennes, France; Comprehensive Cancer Center Eugene Marquis (CCC), Avenue de la Bataille Flandres-Dunkerque, 35042 Rennes Cedex, France; Clinical Investigation Center, University Hospital of Rennes, 2 rue du Professeur Léon Bernard, 35043 Rennes Cedex, France

**Keywords:** Prostatic neoplasms, Randomized controlled trials as a topic, Patient selection, Decision support systems, Clinical, Patient pre-screening

## Abstract

**Background:**

The performance of randomized controlled trials (RCTs) is often hindered by recruitment difficulties. This study aims to explore the pre-screening phase of four prostate cancer RCTs to identify the impact of a systematic pre-selection of eligible patients for RCT recruitment.

**Methods:**

The pre-screening of four RCTs opened at the Comprehensive Cancer Center in Rennes was analyzed retrospectively (French Genitourinary Tumor Group (GETUG) 14, 15, 16, and 17). Data were extracted from electronic multidisciplinary cancer (MDC) reports and manually completed by physicians and medical secretaries. These data were the main source of information for clinicians to discuss treatment alternatives during MDC sessions. The pre-screening decisions made by the clinicians during these MDC meetings were compared with those made after a systematic review of the MDC reports by a clinical research assistant (CRA). Any inconsistencies in decisions between the CRA and the MDC physicians were corrected by the principal investigator (PI).

**Results:**

The pre-screening rate was 9.1% during the MDC meetings, while it was estimated to be 12.9% after the final review by the PI, and 29% after the systematic review by the CRA. The study showed that 77% and 67% of the MDC reports did not mention clinical and pathological Tumor, lymph node and metastasis classification of malignant tumors (TNM) staging, respectively, and that 35 of the CRA’s 47 proposals rejected by the PI concerned implicit information (not specified in the MDC reports). Only one patient was proposed by the PI, and none by the CRA.

**Conclusions:**

These results confirm that pre-screening could be improved by a systematic review of the medical reports. They also highlight the fact that missing data in electronic MDC reports leads to over-enrollment of non-eligible patients, but not to over-exclusion of eligible patients. Thus, our study confirms the potential gain in using semi-automated pre-selection of MDC reports, in order to avoid missing out on patients eligible for RCTs.

**Trial registration:**

The trials evaluated in this study were previously registered with clinicaltrials.gov (registration number: NCT00104741 on 3 March 2005; NCT00104715 on 3 March 2005; NCT00423475 on 16 January 2007; and NCT00667069 on 24 April 2008).

## Background

The randomized controlled trial (RCT) is the gold standard for evaluating the effects of healthcare procedures, but the performance of clinical RCTs is often hindered by recruitment difficulties [1]. Inadequate recruitment causes delays, increases costs, reduces the statistical power of analysis and finally, may lead to failed clinical trials.

Clinical trials are an important option of care for cancer patients since they often provide the most up-to-date and effective treatment for end-stage disease. It has also been suggested that more participation in oncology clinical trials could lead to improved survival in cancer patients [2,3]. Nevertheless, patient enrollment remains particularly low in oncology clinical trials (according to the National Cancer Institute, less than 5% of adults with incident cancer participate in clinical trials in the United States) [4,5].

Barriers to recruitment for oncology clinical trials can be summarized into three main categories [6,7]:

- Individual barriers (such as patients feeling uncomfortable with experimentation or experiencing a loss of control over decision-making, and physician time and effort required for recruitment),
- Barriers related to RCT designs (overly strict or non-adapted criteria, too large a number of RCTs, RCT complexity leading to difficulties in explaining trials to patients, and so forth), and
- Barriers caused by system and organizational features (lack of staff and research nurses, communication difficulties, planning of local resources according to RCT designs, and so forth).

Different strategies, often timesaving and cost-effective, can be used to overcome these barriers (for example, hiring a research nurse or a data manager). But there is also evidence that Health Information Systems and Information Technologies can improve the recruitment process. A review of recent literature shows that many studies examine, discuss, and/or deal with support systems for clinical trial recruitment [5]. These are mainly concerned with addressing individual barriers, and sometimes with the development of tools to ease usability of eligibility criteria. However, the authors highlight the lack of evaluation and workflow integration of the support systems into the RCT recruitment process, and they emphasize the importance of the pre-screening phase for recruitment, particularly for RCT recruitment systems.

In this study, we hypothesized that the improvement of pre-screening of patients eligible for RCTs would lead to an improvement in overall patient enrollment in clinical trials. Thus, the study focused on the pre-screening steps of patient enrollment to RCTs.

The main objective of the present study was to assess the impact of semi-automated decision support systems in improving patient pre-screening in oncology RCTs. Therefore, the study compared the pre-screening decisions made prospectively by the physicians during patient care, with the pre-screening decisions made with a systematic and retrospective review of the medical reports. Then, the study identified factors that could make pre-screening more efficient.

## Methods

### Workflow of multidisciplinary cancer meetings

**Figure 1:**
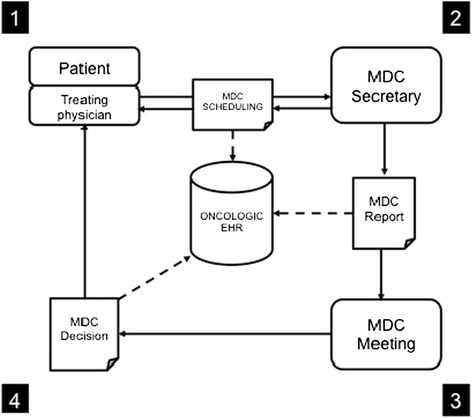
**The therapeutic decision-making process for patients with cancer in Brittany (France), including the multidisciplinary cancer (MDC) meeting.** Step one: MDC meeting request by a treating physician concerning a patient; step two: MDC meeting scheduled by the MDC secretary; step three: presentation of the MDC report during the MDC meeting; step four: registration of the therapeutic decision in the oncologic electronic health records (EHR) and feedback to the treating physician.

As presented in Figure 1, multidisciplinary cancer (MDC) meetings (MDMs) are the start of patient management in oncology and often the first step leading to patient enrollment in cancer RCTs, known as the pre-screening phase.

In Brittany (France), the workflow starts with a request made by the treating physician to the MDC secretary, in order to schedule the presentation of the patient’s case within the MDM (steps one and two). With the patient data provided by the treating physician (most of the time from the paper-based patient record), the secretary fills in an online standard MDC report designed for each type of cancer. The MDC report is intended to collect all essential patient data to make the decision during the MDM.

On the day of the MDM, the secretary shows each MDC report to the tumor board (step three). After the discussion, the therapeutic decision is registered (step four) and sent back to the treating physician who can then inform the patient.

During the MDM, some patients may be proposed (in a pre-screening phase) as being potentially eligible for an RCT; according to a subset of selection criteria called that we will call ‘pre-screening criteria’. These criteria correspond to those that can be checked from the retrospective data available in the patient charts. Pre-screening criteria include, for instance, demographic characteristics (such as age and gender) and some medical characteristics (such as tumor stage and surgical history). The other criteria (which we will call ‘screening criteria’) are those that can only be tested secondarily (in a screening phase), after having contacted the patient (for example, a negative HIV test on the day of enrollment). The patients are eventually included in the appropriate RCT when all eligibility criteria are met.

The present study proposes to retrospectively analyze the decision-making process of the MDM and to compare it to a systematic review of the patient’s case.

### Focus on the ongoing randomized controlled trials used for the study

Urologic oncology was chosen as the experimental field for practical reasons, partly because most eligibility criteria are readily handled by computerized data (Tumor, lymph node and metastasis classification of malignant tumors (TNM) staging, the Gleason score, prostate-specific antigen (PSA) level, and so forth). The study focused on prostate cancer, which is the most common form in urology. It is also the main topic of discussion at urology MDMs and of many clinical trials.

Four prostate cancer clinical trials were active and recruiting patients at the Cancer Research Institute of Rennes during the study period (between October 2008 and March 2009), all conducted by the French Genitourinary Tumor Group (GETUG) [8]:

- GETUG 14, comparing triptorelin, flutamide, and external-beam radiation therapy to external-beam radiation therapy alone in treatment of patients with stage II or stage III prostate cancer (Clinicaltrials.gov identifier: NCT00104741);
- GETUG 15, comparing hormone therapy and docetaxel to hormone therapy alone in treatment of patients with metastatic prostate cancer (Clinicaltrials.gov identifier: NCT00104715);
- GETUG 16, comparing radiation therapy and goserelin to radiation therapy alone in treatment of patients who have undergone surgery for recurrent or refractory prostate cancer (Clinicaltrials.gov identifier: NCT00423475);
- GETUG 17, comparing immediate triptorelin and radiation therapy after surgery to the delayed treatment of patients who have undergone surgery for intermediate-risk stage III or stage IV prostate cancer (Clinicaltrials.gov identifier: NCT00667069).

Detailed eligibility criteria for the four RCTs discussed at the MDMs during the study period are presented in Table 1. The RCTs were conducted in accordance with the Declaration of Helsinki. All participants were required to give written informed consent before enrollment in one of the four studies.

**Table 1:** **Eligibility criteria of GETUG 14, GETUG 15, GETUG 16, and GETUG 17 clinical trials**

	**Eligibility criteria**	**GETUG 14 (** **NCT00104741** **)**	**GETUG 15 (** **NCT00104715** **)**	**GETUG 16 (** **NCT00423475** **)**	**GETUG 17 (** **NCT00667069** **)**
**Disease characteristics**	**Histology**	Histologically confirmed prostate cancer: (Stage T1b-T1c AND PSA ≥10 ng/mL) OR (Stage T1b-T1c AND Gleason score ≥7 OR Stage T2a-T3a)^a^	Histologically confirmed prostate adenocarcinoma^a^	Histologically confirmed prostate adenocarcinoma: pT2, pT3, or pT4, pN0 or pNx^a^	Histologically confirmed prostate adenocarcinoma: pT3a, pT3b (or pT4 by reaching the bladder neck), or R1 disease, OR pN0 or pNx^a^
	**Disease’s prior treatment**	Not specified	Not specified	Treated with surgery only^a^	Treated by curative surgery in the past 6 months^a^
					Positive margins (tumoral glands in contact with contour ink)
	**Metastasis**	No metastatic disease (M0) confirmed by thoracic radiography and bone scan^a^	Metastatic disease^a^	Not specified	Not specified
	**Disease’s exclusion criteria**	History of invasive cancer^a^	Brain metastases^a^	Clinical signs of progressive disease	pN1 disease, pT2 disease,
		Lymph node invasion (N0 or N-)^a^			Other histology than adenocarcinoma^a^
	**PSA/GLEASON score**	PSA <30 ng/mL	Not specified	PSA ≤0.1 ng/mL after prostatectomy^a^	PSA ≤0.1 ng/mL after prostatectomy^a^
				PSA ≥0.2 ng/mL and <2 ng/mL at study entry	Gleason score <8^a^
**Patient characteristics**	**Age**	Under 75^a^	18 and over^a^	18 and over^a^	18 and over^a^
	**Performance status**	ECOG 0-1^a^	ECOG 0-2^a^	ECOG 0-1^a^	ECOG 0-1^a^
	**Life expectancy**	At least 10 years^a^	At least 3 months^a^	≥10 years^a^	≥10 years^a^
	**Hematopoietic**	Not specified	WBC ≥2,000/mm^3^	Not specified	Not specified
			Absolute neutrophil count ≥1,000/mm^3^		
			Platelet count ≥100,000/mm^3^		
	**Hepatic**	Not specified	Bilirubin ≤1.5 × upper limit of normal (ULN) AND AST, ALT ≤1.5 × ULN	Not specified	Not specified
	**Renal**	Not specified	Creatinine ≤150 μmol/L	Not specified	Not specified
	**Cardiovascular**	Not specified	No symptomatic coronary disease^a^	No uncontrolled hypertension	No uncontrolled hypertension
			No congenital cardiac insufficiency^a^	(systolic ≥160, diastolic ≥90 mm Hg)	(systolic ≥160, diastolic ≥90 mm Hg)
			NYHA class < III or IV^a^		
	**Other diseases**	Not specified	No other malignancy (in the past 5 years)^a^	No other malignancy (in the past 5 years)^a^	No other malignancy (in the past 5 years)
			No active infection^a^	No known pituitary gland adenoma^a^	No known hypersensitivity to gonadotropin-releasing hormone^a^
			No severe peripheral neuropathy^a^		No contraindication of intramuscular injection^a^
	**Other**	Not specified	No compliance and follow-up difficulties due to familial, social, geographical, or psychological situation^a^	No compliance and follow-up difficulties due to familial, social, geographical, or psychological situation^a^	No compliance and follow-up difficulties due to familial, social, geographical, or psychological situation^a^
				Affiliated with social security program^a^	Affiliated with social security program^a^
					No patients who are deprived of liberty or under guardianship^a^
**Prior concurrent therapy**	**Chemotherapy**	Not specified	No prior chemotherapy for metastatic prostate cancer (within the past year)^a^	Not specified	Not specified
	**Endocrine therapy**	No prior hormonal therapy^a^	Prior hormonal therapy within the past 2 months allowed^a^	No prior hormonal therapy^a^	No prior hormonal therapy^a^
	**Radiotherapy**	No prior pelvic radiotherapy^a^	No prior radiotherapy to metastatic sites (within the 4 last weeks) ^a^	No prior pelvic radiotherapy^a^	No prior radiotherapy within 3 months after radical prostatectomy^a^
					No prior pelvic radiotherapy^a^
	**Surgery**	No prior radical prostatectomy^a^	No prior surgical castration^a^	No prior surgical or chemical castration^a^	No prior surgical or chemical castration^a^
		No prior castration		At least 6 months since surgery for biological recurrence^a^	
	**Other**	Not specified	No concurrent investigational drugs^a^	No concurrent anticancer therapy^a^	No concurrent participation in another study^a^

^a^eligibility criterion available for pre-screening; pTNM: pathological Tumor Nodes and Metastasis staging; PSA: Prostate-specific antigen; ECOG: Eastern Cooperative Oncology Group (ECOG) Performance status; WBC: White Blood Cell; ULN: Upper limit of normal; AST: aspartate aminotransferase (i.e. SGOT: serum glutamic oxaloacetic transaminase); ALT: alanine aminotransferase (i.e. SGPT: serum glutamic-pyruvic transaminase); NYHA: New York Heart Association (NYHA) Functional Classification

### Data collection

**Figure 2:**
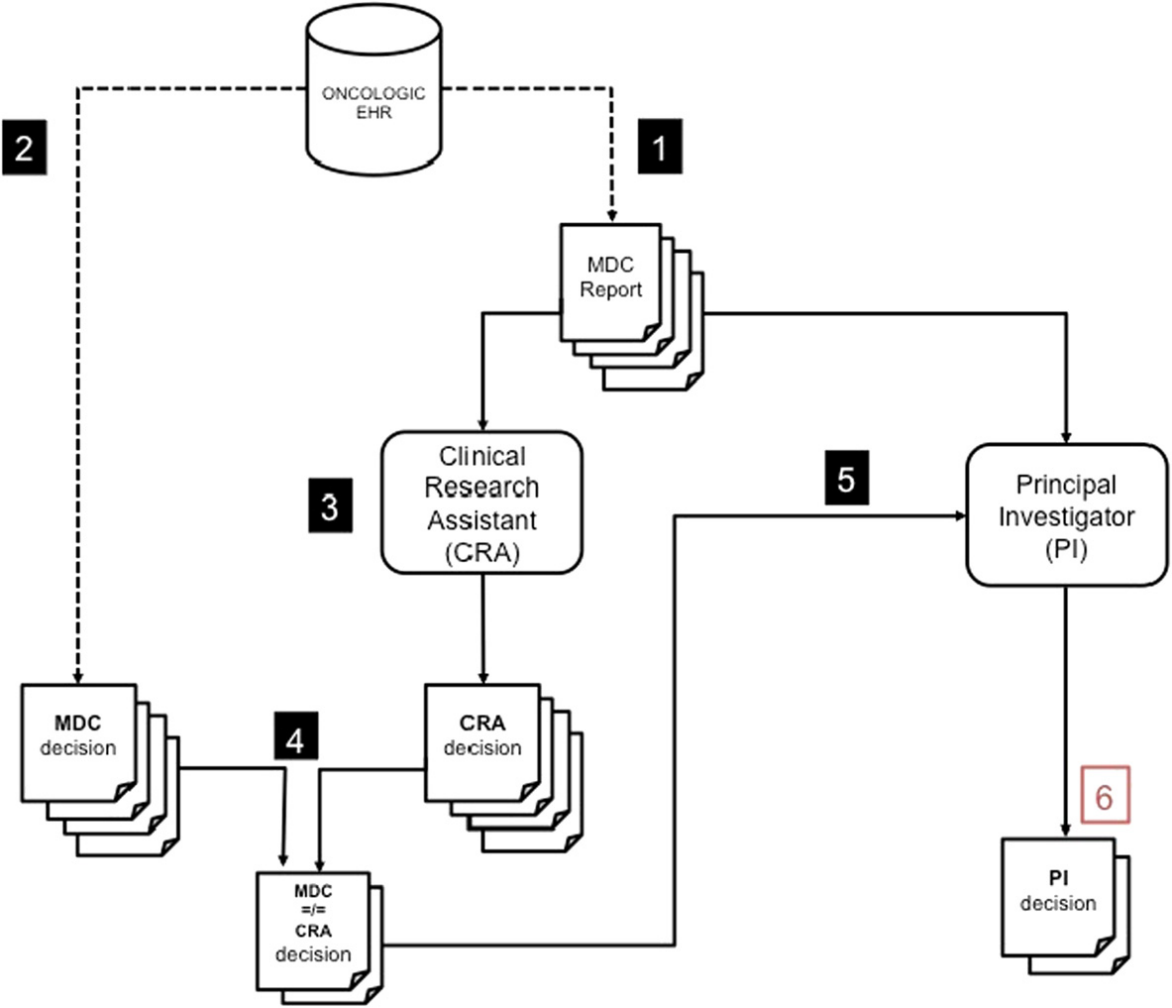
**Evaluation design of the multidisciplinary cancer (MDC) team’s pre-screening decisions.** Step one: extraction of the MDC reports from the oncologic electronic health records (her); step two: Extraction of the MDC team’s pre-screening decisions from the oncologic EHR; Step 3: systematic review of the MDC reports by the clinical research assistant (CRA); step four: comparison of the pre-screening decisions made by the CRA and the MDC team; step five: principal investigator’s review of the MDC reports corresponding to the discrepancies between the CRA’s decisions and the MDC team’s decisions; step six: final pre-screening decisions (gold standard) including the principal investigator’s decisions.

The data used in our study consisted of the information contained in the MDC reports, and of the corresponding therapeutic decisions taken during the MDMs. The MDC reports included general characteristics of patients, medical history, and data related to prostate cancer, such as the Gleason score, tumor staging (clinical and pathological TNM classification), and the prostate-specific antigen (PSA) level. According to each case, the MDC team’s decisions were either to propose a standard therapy to the patient, or to participate in a clinical trial (in this case, the MDC team had estimated that pre-screening criteria seemed to be verified). MDC reports and MDM decisions were retrospectively and exhaustively collected. The data extraction (Figures 1 and 2) corresponded to a period of six months while patient recruitment for the four prostate cancer RCTs was occurring.

Data were completed with the number of medical records and the number of prostate cancer records discussed at each MDC meeting, as well as the number of physicians who participated in the MDMs.

### Ethical statement

The local ethics committee, ‘Comité d’éthique du CHU de Rennes’, approved the present study (registration identifier: 13–26). All four trials cited in this study obtained ethical approval by a research ethics committee with the following approval numbers for GETUG 14: 2003/015 (CPPRB Haute Normandie), for GETUG 15: 04/08 (CPPRB Marseille 1), GETUG 16: 06/025 (CPPRB Lyon), GETUG 17: 2007/53 (CPP Sud-Ouest et Outre Mer III).

### Evaluation and statistical analysis

The evaluation protocol consisted in collecting and comparing three types of decision (MDC, clinical research assistant (CRA), and principal investigator (PI)). The MDC team’s decision was the initial tumor board’s decision made directly during the MDM.

The CRA’s decision (Figure 2, step three) was obtained by asking a CRA experienced in recruitment to clinical trials to systematically review all the MDC reports (while ensuring to hide the MDC team’s decision). Thus, the CRA only had access to information explicitly contained in the MDC reports; the CRA did not have any knowledge or information about the patients other than MDC reports and was unaware of MDC decisions. The MDC decisions and CRA’s decisions were compared (Figure 2, step four). A PI reviewed the MDC reports corresponding to the discrepancies between the CRA’s decisions and MDM decisions (Figure 2, steps five and six). In our study, we considered the PI’s decision (Figure 2, step six) as being the best gold standard as the PI is the person the most involved in clinical trial recruitment.

Results were described using means associated with standard deviations (*m ± ε*), and absolute and relative frequencies (n (%)). The MDC, CRA, and PI pre-screening decisions were described especially by the MDC, CRA and PI pre-screening rate, and compared using the McNemar’s chi-square test. Factors that could influence the MDC team’s pre-screening decisions (the characteristics of the MDMs and the content of the MDC reports) were tested using the Pearson’s correlation test and the Mann–Whitney U test.

A descriptive analysis of the discrepancies between the MDC, CRA, and PI proposals was also performed. Statistical analysis was performed using R software, R Foundation for Statistical Computing, Vienna, Austria [9]. Two-tailed *P* values were reported with *P* <0.05 considered to be statistically significant.

## Results

### Description of the multidisciplinary cancer meetings and the multidisciplinary cancer reports

During the study period, 23 urologic MDMs were held at the Comprehensive Cancer Center of Rennes, and 466 MDC reports were analyzed. Among them, 286 MDC reports concerned patients with prostate cancer, all of which were evaluated in the present study.

On average, 7 ± 1.7 physicians, including 3 ± 0.9 oncologists, attended each MDM. The PI of GETUG 14 was present at 12 of the 23 MDMs held during the study period, the PI of GETUG 15 at 15 of the 23 MDMs, the PI of GETUG 16 at 12 of the 23 MDMs, and the PI of GETUG 17 at 13 of the 23 MDMs.

On average, during an MDM, 20 ± 6.2 reports were discussed with 12 ± 4.6 of those being related to patients with prostate cancer. Clinical and pathological TNM stages were found in 67 (23.4%) and 94 (32.9%) of the 286 MDC reports, respectively concerning patients with prostate cancer. However, 261 (91.3%) and 252 (88.1%) of the 286 MDC reports included the Gleason score and the PSA level, respectively.

### Factors influencing the MDC team’s pre-screening decisions in GETUG 14, 15, 16 and 17

A total of 26 patients were proposed for screening by the physicians who participated in the MDMs; 17 for screening in GETUG 14, seven for screening in GETUG 16 and two for screening in GETUG 17. No patient was proposed for screening in GETUG 15.

Among factors that may have influenced pre-screening during the MDMs (the MDC team’s decisions), a statistically significant and negative correlation was found between the number of MDC reports concerning patients with prostate cancer and the pre-screening rate of each MDM (Pearson correlation coefficient (r) = −0.42, *P* = 0.046). There was also a reverse correlation between the overall number of MDC reports discussed in MDMs and the pre-screening rates of the MDMs, but this negative correlation was not found to be significant (r = −0.37, *P* = 0.082). The number of physicians and the number of oncologists attending the MDMs did not significantly influence the pre-screening rates (*P* = 0.521 and *P* = 0.964, respectively). Neither did the presence of the PI, whatever the RCT (GETUG 14: *P* = 0.095, GETUG 15: no initial pre-screening proposal, GETUG 16: *P* = 0.550, GETUG 17: *P* = 0.950).

Absence of (clinical and pathological) TNM staging, the Gleason Score, or the PSA level in the MDC reports did not hinder MDM attenders to make pre-screening decisions, and did not significantly influence the pre-screening rate of the MDC team (data not shown).

### Pre-screening rates based on the multidisciplinary cancer team’s, clinical research assistants, and PI’s decisions

In total, 146 cumulative MDC report selections were recorded, concerning 86 different patients eligible for at least one RCT. GETUG 14 was the RCT with the most selections during the pre-screening period, with 106 (72.6%) proposals concerning 60 (69.8%) different patients. Pre-screening rates related to each RCT, according to the MDC team’s, CRA’s, and PI’s decisions, are reported in Table 2. The overall pre-screening rates were estimated at 9.1% (26 patients) based on the MDC decisions, 29.0% (83 patients) based on the CRA’s decisions, and 12.9% (37 patients) based on the PI’s decisions.

**Table 2:** **Results of pre-screening of patients eligible for the clinical trials GETUG 14, GETUG 15, GETUG 16, and GETUG 17**

	**GETUG 14**	**GETUG 15**	**GETUG 16**	**GETUG 17**	Total*
MDC team’s decisions (1)	17 (5.9%)	0	7 (2.4%)	2 (0.7%)	26 (9.1%)
CRA’s decisions (2)	59 (20.6%)	8 (2.8%)	13 (4.5%)	3 (1.0%)	83 (29.0%)
PI’s decisions (3)	30 (10.5%)	0	6 (2.1%)	1 (0.3%)	37 (12.9%)

*McNemar’s chi-squared test: (1) versus (2) *P* <10^−3^; (1) versus (3) *P* = 0.022; (2) versus (3) *P* <10^−3^.

(1) Decisions of the physicians during the multidisciplinary cancer (MDC) meetings; (2) Decisions of a clinical research assistant (CRA) after the systematic review of the MDC reports; (3) Final decisions after the principal investigator (PI) corrected the discrepancies between the MDC team’s and the CRA’s decisions.

A total of 22 patients were found eligible for screening in an RCT based on at least two pre-screening methods; most pre-selections were for the same RCTs. Only one discrepancy was observed between decisions made at the MDM and the CRA (GETUG 17 versus GETUG 16, respectively), but the PI retained neither.

**Figure 3:**
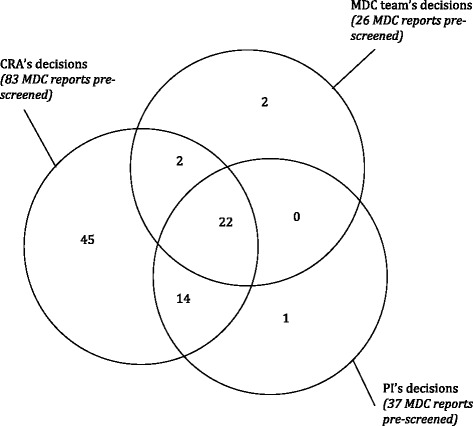
**Venn diagram of the pre-screening of patients eligible for four randomized clinical trials carried out at the Comprehensive Cancer Center in Rennes (GETUG 14, 15, 16, and 17).** Pre-screening was based on the physicians’ decisions during the multidisciplinary cancer meetings (MDC team’s decisions), the decisions of the clinical research assistant (CRA’s decisions), and the principal investigator (PI’s decisions) after a systematic review of the MDC reports.

Discrepancies mainly concerned patients initially excluded during the MDC meetings (59 patients were proposed by the CRA and 14 were finally validated by the PI). Regarding patients initially proposed for an RCT during the MDC meetings, two patients were secondarily excluded by the CRA and, finally, the PI definitively excluded four patients. There was no patient who was not proposed by the CRA but was proposed either during an MDM or by the PI. Nevertheless, the PI proposed one patient who was not proposed either by the CRA or at the MDC meetings. Figure 3 summarizes these results in a Venn diagram.

The four MDC team’s pre-selections excluded by the PI were due to mistakes on the evaluation of some exclusion criteria during the MDMs (too high a PSA rate or the presence of a positive margin after surgery). Among the CRA's 47 pre-selections that were not retained by the PI, 35 were due to implicit information known only by the clinicians and not explicitly available in the MDC reports. For example, the PI excluded 18 CRA proposals because the patients had a Gleason score of less than seven, but such a criterion was not described in the exclusion or inclusion criteria of the RCTs. In the same way, six patients could have been eligible for an RCT but the MDC teams judged another type of care more effective. Further, nine patients were excluded because they had some specific characteristics (vital status, combination of comorbidities, and wishes) not compatible with enrollment in the RCTs according to the MDC teams.

## Discussion

In this study, the pre-screening rate with MDC team’s decisions made during the MDMs was estimated to be at 9.1%. It is higher than rates previously reported in other studies (around 5% for oncology RCTs) [4]. Nevertheless, the best pre-screening rate was estimated to be 12.9% (based on the PI’s decisions). This confirms that patient pre-screening in clinical cancer research could be improved by a systematic review of the MDC reports after MDMs.

It has been shown that individual barriers, such as the time and effort required for patient recruitment for the physicians, are factors that decrease enrollment in RCTs [6]. The present study found that all patients proposed by the PIs were also proposed by the CRA’s systematic review, except one. In other words, the CRA’s review resulted in the justified rejection of more than 200 of the 286 initial MDC reports. Thus, we argue that the addition of a manual (or a computerized) systematic review at the first step of the screening process (the pre-screening phase) could lead to substantial gains in time and effort for the physicians’ screening task afterwards.

Nevertheless, the results showed that the pre-screening decision should not be based only on the information available in the MDC reports, since some data are missing (such as TNM staging), or are not explicit. These issues illustrate why the pre-screening rate (29.0%) is so high when the CRA makes the decision alone; because their decision relies only on the content of the MDC reports. Missing data cannot be used to exclude the patients who are then selected as being eligible for RCTs. From the perspective of automating this decision-making process using a recruitment support system, we can foresee that such a system will overestimate the number of patients potentially eligible for RCTs, just as the CRA has done.

It is likely that the best approach for these systems is not intended to replace the physicians’ decision, but to help physicians by reminding them that every patient is potentially eligible for an available clinical trial. The main objective will therefore be more to avoid missing out on patients who could be included than deciding which patients should be included.

Indeed, our result showed that clinical TNM staging and pathological TNM staging are missing in 68% and 76%, respectively, of the MDC reports proposed by pre-screening after correction by the PI (data not shown). From a decision-making perspective, missing and implicit data (only known by the physicians of the MDC teams) could be seen as problematic regarding patient selection, but our results showed that missing and implicit information usually led to the exclusion of patients. Thus, these results showed that missing and implicit data mainly caused an overestimation of patients eligible for RCTs, resulting in the decreased precision of the systematic (or automated) pre-screening, but this did not lead to the wrongful exclusion of eligible patients.

It could be argued that observations in the present study reflect local patterns of pre-screening practice and medical record design. Indeed, centers with routine systematic pathology reporting, in line with international standards, could certainly have a better registration rate of clinical and pathological TNM staging and thus increase the performance, and above all precision, of systematic (or automated) pre-screening.

This illustrates that the best pre-screening practices cannot be guaranteed without providing the essential information (such as TNM staging) at the time of pre-screening, but they are not limited due to the systematic (or automated) pre-screening methods themselves. The present study shows that despite the presence of numerous missing data concerning key elements for enrollment in RCTs in urologic oncology, the implementation of a manual (or a computerized) systematic review could optimize the pre-screening task by improving the pre-screening rate and by reducing the time and effort required for pre-screening by physicians.

We did not succeed in discovering which factors could really impact the pre-screening rate, except for the number of MDC reports discussed per session. The low number of patients who are actually eligible in the present study could be a limit to the interpretation of such a result, but it confirmed at least that the workload during the MDC meetings is a major factor that decreases pre-screening performance and enrollment in RCTs in oncology. Therefore, this is a strong argument for research as investigated in the automatic selection of clinical trials based on eligibility criteria (ASTEC) project [10], which aims to provide tools to physicians (oncologists) to make the recruitment of patients for RCTs easier and more automatic.

## Conclusions

The study found that a systematic review of medical reports could increase the cumulative pre-screening rates of four RCTs recruiting patients at the Comprehensive Cancer Center in Rennes from 9 to 13%. Also, we argue that such an additional systematic review during the first step of the screening process could lead to substantial gains in time and effort for physicians in their task of recruiting patients for RCTs.

The study also showed that missing data in the MDC reports leads to a systematic review that over-includes non-eligible patients, but does not over-exclude eligible patients. Therefore, we confirm the potential gain in using the semi-automated selection of electronic MDC reports to avoid missing out on patients eligible for RCTs.

To conclude, optimizing the pre-screening process represents an interesting way to improve patient enrollment in clinical trials, which remains particularly low in oncology research. The use of computerized pre-screening support systems to perform a systematic review of eligible patients could help to reach such an objective.

## Abbreviations

ASTEC: Automatic selection of clinical trials based on eligibility criteria
CPP/CPPRB: French research ethics committee
CRA: Clinical research assistant
EHR: Electronic health records
GETUG: French Genitourinary Tumor Group
MDC: Multidisciplinary cancer
MDM: Multidisciplinary cancer meeting
PI: Principal investigator
PSA: Prostate-specific antigen
RCT: Randomized controlled trial
TNM: Tumor, lymph node and metastasis classification of malignant tumors
